# Synthesis and Characteristic of Xylan-grafted-polyacrylamide and Application for Improving Pulp Properties

**DOI:** 10.3390/ma10080971

**Published:** 2017-08-20

**Authors:** Gui-Bin Xu, Wei-Qing Kong, Chuan-Fu Liu, Run-Cang Sun, Jun-Li Ren

**Affiliations:** 1 State Key Laboratory of Pulp and Paper Engineering, South China University of Technology, Guangzhou 510640, China; 13570510843@163.com (G.-B.X.); kongweiqing1119@gmail.com (W.-Q.K.); chfliu@scut.edu.cn (C.-F.L.); 2 Beijing Key Laboratory of Lignocellulosic Chemistry, Beijing Forestry University, Beijing 100083, China; rcsun3@bjfu.edu.cn

**Keywords:** xylan, the graft copolymerization, structure, characteristics, paper properties

## Abstract

Recently, more attentions have been focused on the exploration of hemicelluloses in the paper industry. In this work, xylan-grafted-polyacrylamide (xylan-g-PAM) biopolymers were synthesized by the graft copolymerization of xylan with acrylamide, and their interaction with fibers was also investigated to improve waste newspaper pulp properties with or without cationic fiber fines. The influences of synthesis conditions were studied on the grafting ratio and the grafting efficiency of biopolymers. Prepared biopolymers were characterized by FTIR, ^13^C NMR, TGA and rheology. It was found that the grafting of PAM on xylan was conductive to improve xylan properties, such as the solubility in water, rheological features, and thermal stability, and the maximum grafting ratio was achieved to 14.7%. Moreover, xylan-g-PAM could obviously enhance the mechanical properties of waste paper pulps. Xylan-g-PAM also played the dominant role in increasing the strength of paper in the combination with prepared cationic fine fibers. When the amounts of xylan-g-PAM and cationic fiber fines were 1.0 wt % and 0.5 wt %, the mechanical properties such as the tensile index was increased by 49.09%, tear index was increased by 36.54%, and the burst index was increased by 20.67%, when compared with the control handsheets. Therefore, xylan-g-PAM as the new biopolymer could be promising in the application of strength agents for the paper industry as well as cationic fiber fines.

## 1. Introduction

Hemicelluloses, as the second most common polysaccharides in nature after cellulose, account for 20–35% of lignocellulosic biomass [1,2,3]. As the most abundant hemicelluloses in hardwoods, xylan, is also abundant in agricultural crops such as straw, corn stalks, and hulls [4]. Xylan is composed of a β-1,4-linked D-xylopyranose backbone as proved by a few investigations [4,5,6]. Xylosyl ring of xylan contains two active hydroxyl groups, which provide the opportunity for chemical modification to improve xylan properties for broadening its industrial application. 

Chemical modification plays a very important role in improving the properties of xylan and facilitating its applications. There have been some researches on functionalization of xylan by chemical modification, and application of xylan derivatives. Chemical modification of xylan also broadens the application in preparing films with water resistance [7,8,9], hydrogels with environmental response [10,11,12], paper additives and flocculation aids [13,14] etc. Among the chemical modification methods, less attention has been paid on the graft polymerization of xylan. Carboxymethyl xylan-g-poly (propylene oxide) was prepared to enhance the hydrophobicity for film preparation [15]. Xylan grafted with polyethylene glycol as moisture-absorption/retention biomaterials was obtained in ionic liquids [16]. Hemicelluloses-g-polyacrylamide was prepared in diluted alkaline aqueous solution to improve the solubility in water of the water-insoluble hemicelluloses [17]. Xylan-g-polyacrylonitrile was synthesized by emulsion polymerization to improve the thermal stability [18].

A few literatures refer to the interaction of xylan derivatives with pulp fibers for improving the paper properties. Polyelectrolyte complexes of chitosan and xylan attributed strength improvements of paper to forming a random array of contact points at the solid-liquid interface which could create efficient joints between two treated surfaces, and increase the adhesion between the fibers [19]. The addition of 2-hydroxypropyltrimethylammonium xylan chloride obviously enhanced the tensile index of paper for its self-structuring behavior in solutions and electrostatic interactions, as compared to untreated pulp [14]. The absorption of cationic xylans onto the anionic pulp surface decreased the electrostatic repulsion effect, and therefore enhanced the inter-fiber linkages and mechanical properties [20]. The cationic 4-*O*-methyl glucuronoxylan as the strength wet-end additive could improve the paper strength properties when compared to native paper, including tensile and burst indices, which was attributable to strong fiber-to-fiber bonds [21]. Thus, xylan derivatives increased the strength of pulp fibers by an electrostatic interactions and hydrogen bonds combination, and thus the paper properties were obviously improved [22]. Thus, xylan and their derivatives as strength additives have potential in improving pulp properties.

In the paper industry, some part of fiber fines as an important component of pulp on forming wire were removed into the white water system during the dewatering procedure of the pulp suspension, consequently causing the low efficient utilization of resources. Fiber fines could improve the interaction of fibers by three ways: including bonding strength, the bonding area of the cross-section of the fibers, and the number of combination of fibers [23]. However, fiber fines had the limited ability to improve the pulp properties. Cationic modification could endow fiber fines with cationic groups, which could enhance the interaction of them with pulp fibers. But limited literature has been focused on the cationic modification of fiber fines and their application.

In this work, we prepared xylan-g-polyacrylamide (xylan-g-PAM) biopolymers by the graft copolymerization of xylan with acrylamide (AM) under the initiator of potassium persulfate, and investigated their application for improving the mechanical properties of waste pulp. The influences of synthesis conditions were discussed on the grafting efficiency, and the grafting ratio of biopolymers. The characteristics of chemical structure, thermal stability, and the rheological behavior of prepared products were conducted by Fourier transform infrared spectroscopy (FTIR), carbon nuclear magnetic resonance spectroscopy (^13^C NMR), thermogravimetric analysis (TGA), elemental analysis (EA), and rheology. The biopolymers as the strengthening agent was also studied to improve the mechanical properties of waste newspaper pulp, as well as their work in combination with cationic fiber fines obtained from white water cycles during the process of papermaking for waste newspaper, as well as their coordination work with cationic fine fibers.

## 2. Experimental Section

### 2.1. Materials

Commercial beechwood xylan (Sigma Chemicals, Taufkirchen, Germany, Product No. X0504, ≥90% xylose) was used without further purification. Fiber fines and waste newspaper pulp were obtained from the Guangzhou paper mill. Waste newspaper pulp is mainly composed of American waste paper. AM was purchased from TianJin FuChen Chemical Reagent Factory (Tianjin, China). Potassium persulfate was obtained from Tianjin Damao Chemical Reagent Factory (Tianjin, China). Glacial acetic acid was bought from Guangdong Guanghua Technology Company (Guangzhou, China). Glycol was purchased from Shanghai Lingfeng Chemical Company (Shanghai, China). Acetone was purchased from Nanjing Chemical Company (Nanjing, China). 3-chloro-2-hydroxypropyltrimethylammonium chloride (CHMAC), NaOH, and HCl were purchased from Guangzhou Chemical Reagent Factory (Guangzhou, China). Chemicals used in this study were analytical reagent grade, and were used without any purification. Deionized water was used in all experiments.

### 2.2. Preparation of Xylan-g-PAM

The graft copolymerization of PAM onto xylan was carried out in a 250 mL three-necked round bottom flask. The flask was kept in a water bath maintained at the desired temperature (50–80 °C). Firstly, a certain amount of xylan (1 g) was dissolved in 25 mL of deionized water, and was stirred for 40 min under the protection of nitrogen. 5 mL of potassium persulphate solution of required concentrations (Table 1) was added, and nitrogen was continuously purged for 10 min. Then, 10 mL of required concentrations of acrylamide was added in the xylan solution. The mixture was stirred for 4 h with a slow stream of nitrogen. After the reaction was over, the round bottom flask products was poured into the beaker with stirring and washing with acetone, the precipitate was washed again by acetone for a few times, dried for 24 h in the vacuum drying oven. The crude product (*W*_1_) was obtained and extracted in the mixed solution of glacial acetic acid and ethylene glycol for 12 h, then dried and the refined products (*W*_2_) was obtained. 

Grafting parameters of these biopolymers were determined as below:

The grafting ratio = (*W*_2_ − *W*_0_)/*W*_0_ × 100%

The grafting efficiency = *W*_2_/*W*_1_ × 100%

where *W*_0_ is the weight of xylan; *W*_1_ is the weight of the crude product; and, *W*_2_ is the weight of the refined products.

### 2.3. Preparation of Cationic Fiber Fines

A mixture solution with NaOH/urea/H_2_O (*w*/*w*/*w*, 7:12:81) was precooled to −12 °C. Then the dried fiber fines were immediately dispersed into the solvent system under vigorous stirring for 30 min at the ambient temperature to obtain the transparent solution with a concentrations of 2 wt %, and then the required amount of CHMAC (with a 4 wt % concentration) was added according to the optimal reaction condition of the literature [24]. After the mixture was stirred to form the homogeneous system, the solution was placed in an oven at 60 °C for 3 h. The obtained products were dissolved in distilled water, neutralized with 0.1 M HCl, and then slowly poured into the 80% ethanol to remove unreacted reagents. The purified products were dried in a vacuum oven at 60 °C for 16 h [8].

### 2.4. Characterization of Prepared Products

Element analysis (EA) is a quantitative analysis of the specific elements of the samples. Specimens weighing approximately 3–5 mg were heated in an oxygen atmosphere. The N, C, and H contents of xylan were 0.00%, 38.34%, 6.24%, respectively, while they became 8.85%, 44.23%, and 6.88% after the modification of xylan. The increase in the N content confirmed the presence of nitrogenous compounds (AM) in the modified xylan. The N, C, and H contents of fiber fines were 0.87%, 29.02%, 4.08%, respectively, and after modification, they were 3.59%, 29.1%, 4.99%, indicating that cationic groups were present on the fines fibers.

Xylan and fiber fines after and before modification were dried in the infrared drying oven. FTIR analysis was performed using a Fourier transform spectrophotometer (Nicolet 750, Thermo Fisher Scientific, Waltham, FL, USA) appended Attenuated Total Reflectance technique. A total of 32 scans were accumulated in the transmission mode, with a resolution of 4 cm^−1^. The spectrum was obtained from a range of 4000 cm^−1^ to 400 cm^−1^.

The solution-state ^13^C-NMR spectra were recorded on a Bruker DRX-400 spectrometer (Bruker, Karlsruhe, Germany) at 25 °C after 15,000 scans. The sample (80 mg) was dissolved in 1 mL of D_2_O. A 30° pulse flipping angle, a 9.2 μs pulse width, a 1.36 s acquisition time, and 2 s relaxation delay time were used.

TGA was carried out using thermo gravimetric analysis on a simultaneous thermal analyzer (TGA Q500, TA Instruments, New Castle, DE, USA). About 5 mg of samples were heated to 700 °C from room temperature with the heating rate of 10 °C/min in a nitrogen atmosphere. The apparatus was flushed with nitrogen at a flow rate of 20 mL/min.

Dynamic rheological properties of xylan and xylan-g-PAM were measured by using a sandwich rheometer AR 2000 (TA Instruments, New Castle, DE, USA). Purchased xylan was not dissolved completely in water at room temperature, and thus it was dissolved in the dilute 1 wt % NaOH solution to form the homogeneous solution, and xylan-g-PAM with different grafting ratios was dissolved in water at 25 °C, and stirred to form the stable solution. Then, the solutions were dropped on Brookfield D VIII instrument panel. Setting the parameter and rheometer control and data collection were performed using the manufacturer’s Rheo2000 software provided with the instrument. For all tests, shear rate, frequency, viscosities, the storage modulus (G′), and the loss modulus (G″) were collected.

### 2.5. Preparation of Handsheet Formation and Mechanical Properties Test

Five sheets were prepared by each treatment according to GB 2828-81 norms (Chinese Technical Association of Pulp and Paper). Each sheet has a grammage (weight per unit area) of ~55 g/m^2^. Xylan-g-PAM was added to the waste newspaper pulp during homogenization for 10 min before sheet formation. Then, papermaking was carried out in the fast papermaking machine. The product was placed in a constant temperature and humidity room for 24 h. The samples were cut into the definite shape for the paper performance test.

In addition, the xylan-g-PAM was respectively added to the waste paper pulp with fiber fines and cationic fiber fines, the procedure was followed as the step mentioned above. 

The mechanical properties of handsheets were tested by China norm (Chinese Technical Association of Pulp and Paper). At least 12 tests strips were evaluated for each pulp sample, and the standard deviation was always less than 1.

## 3. Results and Discussion

### 3.1. Influence of the Synthesis Conditions on Xylan-g-PAM

**Figure 1:**
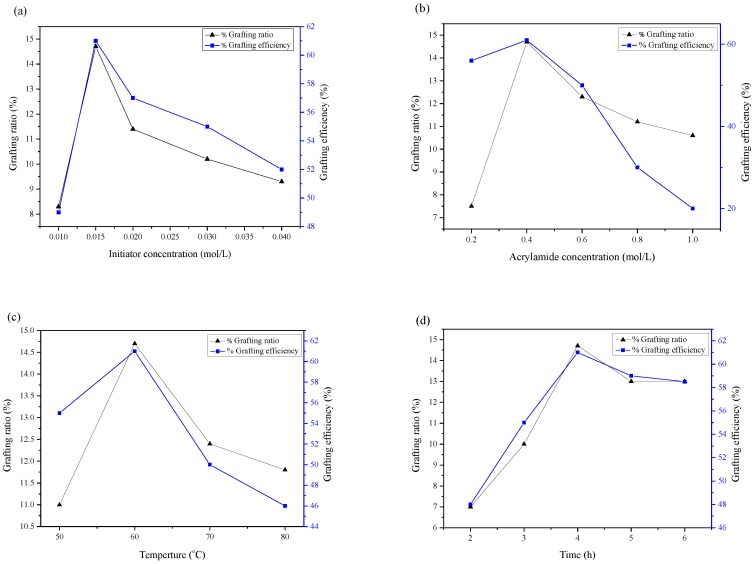
The influences of the synthesis conditions on the grafting ratio and the grafting efficiency of xylan-g-PAM. (**a**) The influence of initiator; (**b**) The influence of acrylamide; (**c**) The influence of temperature; and (**d**) The influence of temperature.

The influences of the initiator concentration on the grafting ratio and the grafting efficiency of xylan-g-PAM were shown in Figure 1. With increasing the initiator concentration, the grafting ratio and grafting efficiency were increased (Figure 1a). When the concentration was 0.015 mol/L, the grafting ratio and the grafting efficiency reached the maximum values, corresponding for 14.7% and 61%, respectively. When the concentration was above 0.015 mol/L, the grafting ratio and the grafting efficiency began to decrease, which was due to the participation of the initiator in the termination step of the growing chains [25]. In addition, more initiator could result in the homopolymerization of PAM.

The effect of the AM concentration on the grafting ratio and the grafting efficiency was illustrated in Figure 1b. The grafting ratio and the grafting efficiency were increased sharply when AM concentration was lower than 0.40 mol/L because the more monomers (AM) reacted with xylan as the AM concentration increased. When the AM concentration was 0.40 mol/L, the grafting ratio reached the maximum values (14.7%). Afterwards, the grafting efficiency and the grafting ratio rapidly decreased. The high AM concentration resulted in the self-polymerization, which led to a decline in the grafting ratio and the grafting efficiency. In Figure 1c,d, when increasing the reaction temperature and time, the grafting ratio and the grafting efficiency firstly increased and then decreased. Higher temperature accelerated the graft copolymerization of xylan with AM. At 60 °C, they reached the maximum values. However, beyond 60 °C, the grafting ratio and the grafting efficiency decreased because the homopolymerization was dominated at a higher temperature [26]. In addition, chain transfer reactions and chain termination may also be increased at a higher temperature, leading to the decrease in the grafting ratio and grafting efficiency [27]. In Figure 1d, when the reaction time was less than 4 h, the grafting ratio and the grafting efficiency increased rapidly as the reaction time increased. When the reaction time was 4 h, the grafting ratio, the grafting efficiency reached the maximum values, corresponding for 14.7% and 61%, respectively. With further prolonging the reaction time, the concentration of monomer and free radicals in the system was decreased, which resulted in the leveling off of the grafting ratio and the grafting efficiency. Moreover, the chemical modification improved the dissolution of xylan in water, and xylan-g-PAM could be dissolved in the water completely to form the transparent solution at room temperature.

### 3.2. FTIR Spectra

**Figure 2:**
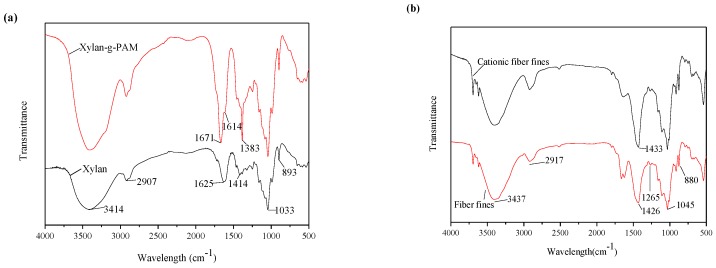
FTIR spectra of xylan and xylan-g-PAM (**a**); fiber fines and cationic fiber fines (**b**).

The FTIR spectra of xylan and xylan-g-PAM were given in Figure 2a, and the FTIR spectra of fiber fines and cationic fiber fines were displayed in Figure 2b. In Figure 2a, the absorbance at 3414 cm^−1^, 2907 cm^−1^, 1625 cm^−1^, 1414 cm^−1^, 1033 cm^−1^, and 893 cm^−1^ in the spectrum are associated with xylan [28]. The band at 2907 cm^−1^ is assigned to the C–H stretching vibration of alkane in xylan. In the spectrum of xylan-g-PAM, the intensities of peak around 3414 cm^−1^ increased due to the superposition of the stretching vibration absorption peak of –OH and –NH. In contrast, two strong bands around 1671 cm^−1^ and 1614 cm^−1^ are attributed to amide-I (C=O stretching) and amide-II (NH bending) [29]. The band around 1383 cm^−1^ is for the C–N stretching vibrations [29]. These results confirmed that the acryloyl groups were introduced to the xylan chains successfully.

In Figure 2b, the absorption at 3437 cm^−1^ corresponds to the stretching of hydroxyl (O–H) groups in fibers. The strong band at 2917 cm^−1^ corresponds to the alkyl (C–H) stretching of vibrations in fibers. The absorbance at 1265 cm^−1^ is attributed to the C–O stretching of acetyl group in hemicelluloses [30]. The small sharp absorbance at 880 cm^−1^ is attributed to the β-glucosidic linkages between the sugar units in hemicelluloses and cellulose [30]. Compared with the spectrum of fiber fines, an enhancement in the intensity of the ether bond absorbance at 1045 cm^−1^ provided the evidence of quaternization. A change appeared in an increase in the intensity of the band at 1433 cm^−1^, assigned to the C–N bending vibration [31]. These results confirmed that the cationic groups were introduced to the fines chains successfully.

### 3.3. TGA Analysis

**Figure 3:**
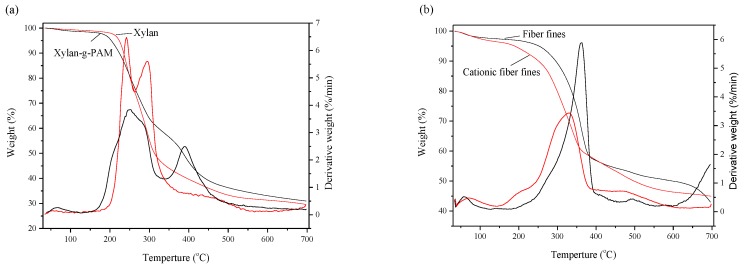
The TGA/DTA curves of xylan and xylan-g-PAM (**a**); fiber fines and cationic fiber fines (**b**).

Figure 3 shows the typical TGA/DTA curves of xylan, xylan-g-PAM, fiber fines, and cationic fiber fines. For Figure 3a, the TG curves of xylan and xylan-g-PAM exhibited a three-stage thermal decomposition. The first stage was below 200 °C, indicating on the surface water evaporation. In the range of 200–400 °C, the substantial weight loss of xylan and xylan-g-PAM occurred, which was attributed to the thermal decomposition of xylan, amide side-groups of grafted AM [32]. During this period, the decomposition rate of xylan was faster than xylan-g-PAM, indicating that xylan-g-PAM was thermally more stable than xylan. In the range of 400–700 °C, the TGA curves of xylan and xylan-g-PAM were smoothness. At 700 °C, the residue weight of xylan was higher than xylan-g-PAM due to the little impurity of purchased xylan (some inorganic salt and ash content). 

For Figure 3b, a small weight loss occurred below 250 °C, implying a loss of moisture present in the samples. In the temperature range of 250–400 °C, the substantial weight loss of fiber fines and cationic fiber fines occurred. Fiber fines and cationic fiber fines showed their T_max_ (the decomposition temperature corresponding to the maximum weight loss rate) at 362 °C and 330 °C, respectively, implying that the cationic modification caused the low thermal stability of fiber fines, due to the fact that the hydrogen bonds and molecular structure were destroyed, and grafted cationic groups were unstable. 

### 3.4. ^13^C-NMR Spectra

**Figure 4:**
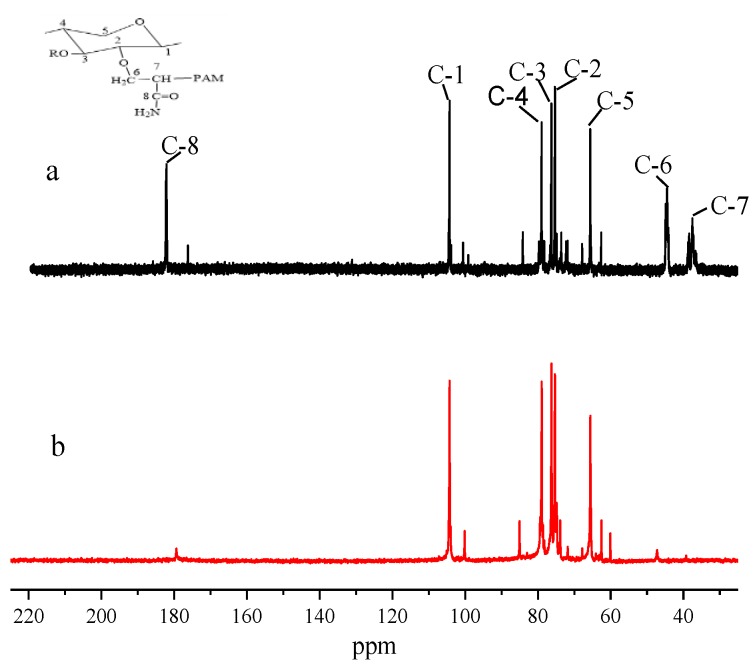
^13^C-NMR Spectra of xylan-g-PAM (**a**) and xylan (**b**).

Xylan and xylan-g-PAM were characterized by ^13^C-NMR spectroscopy in D_2_O to confirm the structural features, as shown in Figure 4. In the spectrum of xylan (spectrum b), the main (1→4)-linked β-d-xylp units were obviously characterized by the signals at 104.3 ppm, 78.9 ppm, 76.3 ppm, 75.3 ppm, and 65.6 ppm, which is attributed, respectively, to C-1, C-4, C-3, C-2, and C-5 of the β-D-xylp units [8]. In the ^13^C-NMR spectrum of xylan-g-PAM (spectrum a), there were great changes in the number and positions of the strong signals, in comparison with xylan. One new signal was present at 37.3 ppm, which was sp^3^ hybridized carbon atoms (i.e., –(CH_2_–CH)n units in the graft copolymer [25]. Peak at 44.3 ppm was assigned to methylenes, which were connected to the C-6. The signal at 181.95 ppm was for the amide carbonyl carbon. This phenomenon clearly showed PAM was grafted onto the xylan chain. Moreover, the intensity of these peaks at C-2 and C-3 on the xylosyl ring of xylan chains was changed, indicating that the hydroxyl groups on C-2 and C-3 positions reacted with PAM. The intensity at the C-2 signal was increased after xylan modification, which implied that more reactions occurred on hydroxyl groups at C-2 position than those at C-3 position of xylan.

### 3.5. Rheological Study

**Figure 5:**
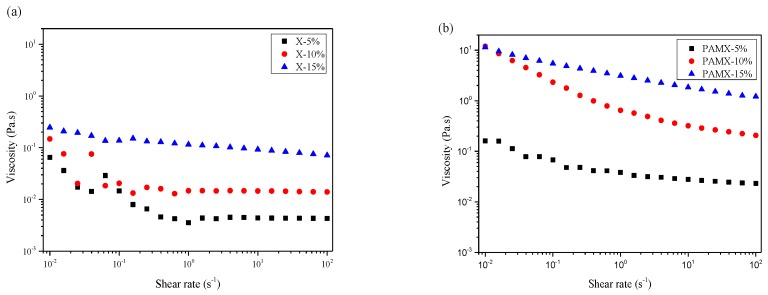
Shear rate dependence of viscosity for xylan (X) (**a**); and xylan-g-PAM (PAMX) (**b**) at different concentrations.

**Figure 6:**
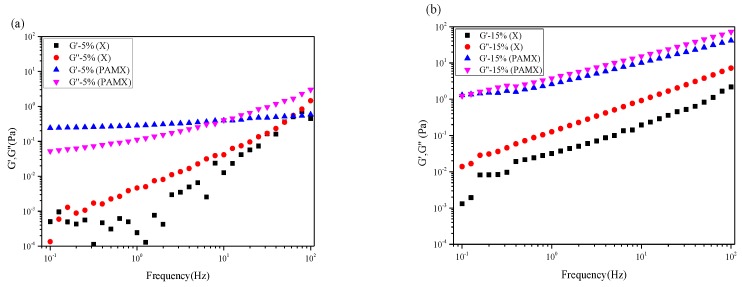
Frequency dependent modulus of the solutions of xylan (X) and xylan-g-PAM (PAMX) at 5% concentration (**a**); at 15% concentration (**b**).

There are few investigations on the rheological behavior of xylan and its derivatives. For better understanding of the physico-chemical properties of biopolymers, the rheological behavior of xylan and xylan-g-PAM was investigated in this study, as shown in Figure 5 and Figure 6. In Figure 5, the viscosity of xylan and xylan-g-PAM decreased with the increasing of shear rate, indicating that these solutions exhibited pseudoplastic or shear-thinning behavior in the range of shear rates tested, due to the destroying of network structure of xylan and its derivatives [33]. The viscosity of xylan-g-PAM solution was higher than that of xylan solution at 5%, 10%, and 15% concentrations in the whole shear rate rang, which suggested that grafting PAM chains on xylan backbone led to shear stable and viscosity-increasing grafted xylan copolymers [34].

Figure 6 shows the rheological properties (storage modulus, G′, and loss modulus, G″) of xylan (a), and xylan-g-PAM (b). In the dynamic rheological test, storage modulus and loss modulus are important parameters. When the storage modulus was increased, the degree of material deformation was smaller under the force. With the loss modulus increasing, it was not easy to flow under the force. At the concentration of 5% (Figure 6a), the storage modulus was lower than the loss modulus over the entire frequency region for the xylan solution, showing a viscous behavior [35]. The xylan-g-PAM solution showed a stronger elastic property where G′ was above G″ in the range 0.1–10 Hz when the concentration was 5%, which was due to the strong molecule entanglement [33]. At the concentration of 15% (Figure 6b), the storage modulus was lower than the loss modulus over the entire frequency region for the xylan-g-PAM solution, indicative of a viscous behavior. The storage modulus and loss modulus of xylan-g-PAM was higher than the xylan in the same concentration in Figure 6. It showed that the solution of xylan-g-PAM exhibited a greater viscosity behavior than the xylan solution.

### 3.6. Influence of Xylan and Xylan-g-PAM on the Mechanical Properties of Handsheets of Waste Newspaper Pulp

Table 2 displays the influence of the different amount of xylan on the mechanical properties of handsheets. Obviously, the mechanical properties of handsheets were increased little when the xylan with different amounts was added. As the amount was 1.0 wt %, as compared to control handsheet, the tensile index was increased by 6.59%, tear index was increased by 8.64%, burst index was increased by 8.67%, and folding time was not obvious. The little improvement was due to less hydrogen bonds forming between xylan and fibers. Thus, xylan could not easily retain fibers and increase the binding force of fibers.

Table 3 shows the influence of xylan-g-PAM with different grafting ratios on the mechanical properties of handsheets. Hydrogen bonds and electrostatic adsorption are the main influence factors for improving the paper strength. When the xylan-g-PAM was added, the acylamino group on xylan-g-PAM and the hydroxyl radical on fibers could form strong intermolecular interaction and hydrogen bonds. Thus it could increase the binding force of fibers in the paper, thereby improving the strength index based on binding force, such as tensile strengthen, tear strengthen, burst strengthen, and folding strengthen [36]. Higher grafting ratio of xylan-g-PAM has more acylamino group, which could form stronger intermolecular interaction and more hydrogen bonds with fibers, consequently causing the stronger paper properties. When the grafting ratio was 14.7%, as compared to the control pulp, the tensile index was increased by 35.54%, the tear index was increased by 49.17%, the burst index was increased by 46.67%, and folding times were increased obviously. The strengthen interaction with fibers was attributed to the form of more connecting bonds among xylan-g-PAM and pulp fibers.

Table 3 also displays the influence of the different amount of xylan-g-PAM on the mechanical properties of handsheets. With the increase in the amount of xylan-g-PAM with the grafting ratio of 14.7%, the mechanical properties of handsheets were improved. When the amount was more than 1 wt %, the performance of paper decreased gradually. The low amount of xylan-g-PAM could improve the mechanical properties of handsheets through retaining fibers and forming hydrogen bond with fibers. However, when the xylan-g-PAM amount was 1.5 wt %, some gel particles were detected on the surface of the handsheets [37]. The mechanical properties of handsheets were decreased due to the reduced effect of the combination of xylan-g-PAM and fibers.

### 3.7. Influence of Xylan-g-PAM with Fiber Fines or Cationic Fiber Fines on the Mechanical Properties of Handsheets

Table 4 shows the influence of the combination of xylan-g-PAM (PAMX) and fiber fines (FF) on mechanical properties of handsheets of waste paper pulp. As shown clearly, both of the simultaneous addition xylan-g-PAM and fiber fines increased mechanical properties of handsheets. The burst index and tensile index of handsheets under the combination were higher than fiber fines only, and were increased by 30.67%, and 35.36%, when compared with that of control handsheets without any additives. This indicated that xylan-g-PAM could further improve the properties of handsheets under the function of fiber fines.

Table 4 also displays the influence of the combination of PAMX and cationic fiber fines (CF) on the mechanical properties of handsheets of waste newspaper pulp. The addition of CF had the significant impact on improving the mechanical properties of handsheets because CF had cationic groups, which could absorb the anionic groups of the pulp fibers to form electrostatic adsorption, and, therefore enhanced the inter-fiber linkages and mechanical properties. Under the combination of CF and PAMX, the higher strength could be achieved, and the tear index and tensile index of handsheets were increased by 36.54% and 49.09%, as compared with that of control handsheets. This obvious improvement was due to the combined effect of hydrogen bonds and electrostatic adsorption, formed among CF, PAMX, and fibers, and PAMX played the dominant role in improving the strength of pulp.

## 4. Conclusions

In conclusion, biopolymers (xylan-g-PAM) were successfully synthesized by the graft copolymerization of xylan with AM under the function of initiator. Their application was also investigated for improving the mechanical properties of waste newspaper pulp with or without fiber fines and cationic fiber fines. Results showed that the great influence of synthesis condition was observed on the grafting ratio of xylan-g-PAM. The grafting ratio reached the maximum value of 14.7% (60 °C, 4 h). PAM grafted on xylan chain could contribute to improving some xylan properties, such as the solubility in water, rheological features and thermal stability. Xylan-g-PAM could obviously improve the pulp properties. The maximum mechanical properties were achieved, which was increased by 35.54% (tensile index), 49.17% (tear index), 46.67% (burst index), respectively, when compared with pulp without any additives. Moreover, cationic fiber fines could also remarkably improve pulp properties. The simultaneous addition of cationic fiber fines and xylan-g-PAM significantly enhanced some properties of pulp, especially for the tensile index, which was increased by 49.09%, as compared with the control handsheets. This improvement may be due to the dominant role of xylan-g-PAM.

## Figures and Tables

**Table 1:** Synthesis conditions of xylan grafted with polyacrylamide.

Number	Temperature (°C)	Monomer Concentration (mol/L)	Initiator Concentration (mol/L)	Time (h)	Graft Ratio (%)	Graft Efficiency (%)
1	60	0.4	0.010	4	8.3	49
2	60	0.4	0.015	4	14.7	61
3	60	0.4	0.020	4	11.4	57
4	60	0.4	0.030	4	10.2	55
5	60	0.4	0.040	4	9.3	52
6	60	0.2	0.015	4	7.5	56
7	60	0.6	0.015	4	12.3	50
8	60	0.8	0.015	4	11.2	30
9	60	1.0	0.015	4	10.6	20
10	50	0.4	0.015	4	11	55
11	70	0.4	0.015	4	12.4	50
12	80	0.4	0.015	4	11.8	46
13	60	0.4	0.015	2	7	48
14	60	0.4	0.015	3	10	55
15	60	0.4	0.015	5	13	59
16	60	0.4	0.015	6	13	58.5

**Table 2:** Influence of xylan with different amounts on the mechanical properties of handsheets.

The Amount of Xylan (wt %)	Tear Index (mN·m^2^/g)	Burst Index (kPa·m^2^/g)	Tensile Index (Nm/g)	Folding Strength (Time)
0	6.02 ± 0.45	1.50 ± 0.11	22.00 ± 0.91	3 ± 0.4
0.3	6.30 ± 0.52	1.52 ± 0.13	20.13 ± 0.74	3 ± 0.4
0.5	6.53 ± 0.48	1.61 ± 0.17	22.57 ± 0.58	3 ± 0.5
1.0	6.54 ± 0.61	1.63 ± 0.15	23.45 ± 0.63	4 ± 0.5
1.5	6.49 ± 0.57	1.59 ± 0.17	23.12 ± 0.70	4 ± 0.5

Note: Quantitative of each paper was approximately 55 g/m^2^, the stirring time of pulp after addition of xylan was 10 min.

**Table 3:** Influence of xylan-g-PAM with different grafting ratios and different amount on the mechanical properties of handsheets.

Grafting Ratio (%)	The Amount of Graft Copolymer (wt %)	Tear Index (mN·m^2^/g)	Burst Index (kPa·m^2^/g)	Tensile Index (Nm/g)	Folding Strength (Time)
0.00	0	6.02 ± 0.45	1.50 ± 0.11	22.00 ± 0.91	3 ± 0.4
8.3	1.0	7.84 ± 0.32	2.15 ± 0.15	27.82 ± 0.77	5 ± 0.6
12.4	1.0	8.25 ± 0.61	2.10 ± 0.23	28.18 ± 0.83	5 ± 0.4
14.7	1.0	8.98 ± 0.55	2.20 ± 0.20	29.82 ± 0.76	6 ± 0.5
14.7	0.3	7.79 ± 0.51	1.57 ± 0.17	24.46 ± 0.98	3 ± 0.4
14.7	0.5	8.10 ± 0.33	1.97 ± 0.13	26.91 ± 0.87	4 ± 0.5
14.7	1.5	7.52 ± 0.38	1.85 ± 0.16	26.30 ± 0.83	4 ± 0.5

Note: Quantitative of each paper was approximately 55 g/m^2^, the stirring time of pulp after addition of xylan-g-PAM was 10 min.

**Table 4:** Influence of the combination of PAMX and FF or CF on mechanical properties of handsheets of waste paper pulp.

Sample	Dosage of FF and PAMX (wt %)	Dosage of CF and PAMX (wt %)	Tear Index (mN·m^2^/g)	Burst Index (kPa·m^2^/g)	Tensile Index (Nm/g)	Folding Strength (Time)
The blank	0	-	6.02 ± 0.45	1.50 ± 0.11	22.00 ± 0.91	3 ± 0.4
1	1.0 + 0	-	8.18 ± 0.57	1.70 ± 0.16	26.46 ± 0.83	4 ± 0.5
2	0.5 + 1.0	-	8.19 ± 0.36	1.96 ± 0.13	29.78 ± 0.73	5 ± 0.4
3	0 + 1.0	0 + 1.0	8.98 ± 0.55	2.20 ± 0.20	29.82 ± 0.76	6 ± 0.5
4	-	1.0 + 0	8.01 ± 0.38	1.87 ± 0.17	29.88 ± 0.87	6 ± 0.5
5	-	0.5 + 1.0	8.22 ± 0.51	1.81 ± 0.16	32.80 ± 0.90	6 ± 0.5

Note: Quantitative of each paper was approximately 55 g/m^2^, the grafting ratio of xylan-g-PAM was 14.7%, the stirring time of pulp after addition of additives was 10 min.
